# Mortality and its predictors among neurosurgical patients admitted to the surgical ICU in a resource-limited setting: experience from Ethiopia

**DOI:** 10.1007/s00701-026-06842-2

**Published:** 2026-03-24

**Authors:** Absera Gebriel Yohannes, Molla Asnake Kebede, Amira Shamil, Kidus Geabriel Yohannes, Dejen Tekiea Gebrewahd, Turi Abateka Abadiga, Abera Kuma, Selemon Gebrezgabiher Asgedom

**Affiliations:** 1 Department of Anesthesiology, School of Medicine, College of Medicine and Health Science, Addis Ababa University, Addis Ababa, Ethiopia; 2 Department of Medicine, College of Medicine and Health Science, Mizan Tepi University, Mizan Aman, Ethiopia; 3 Department of Pediatrics, College of Medicine and Health Science, Mizan Tepi University, Mizan Aman, Ethiopia; 4 Department of Neurosurgery, Ayder Comprehensive Specialized Hospital(ACSH), Mekelle University, Mekelle, Ethiopia; 5 Department of Pharmacy, Mizan Tepi University Teaching Hospital, Mizan Aman, Ethiopia; 6 Department of Neurology, College of Medicine and Health Science, Hawassa University, Hawassa, Ethiopia; 7 School of Medicine, Yekatit 12 Hospital Medical College, Addis Ababa, Ethiopia

**Keywords:** Neurosurgical patients, Intensive care unit, Mortality, Predictors, Ethiopia

## Abstract

**Background:**

Neurosurgical patients frequently require intensive postoperative care. However, data on the factors influencing their outcomes in low-resource environments are limited. This study aimed to identify the predictors of mortality among neurosurgical patients admitted to the Surgical Intensive Care Unit at the Tikur Anbessa Specialized Hospital, Ethiopia.

**Methods:**

A retrospective cohort study was conducted between January 1, 2024, and January 1, 2025. All neurosurgical patients admitted to the surgical Intensive Care during the study period were reviewed. Patient outcomes were categorized as death during Intensive Care Unit stay or discharge or transfer with clinical improvement. Multivariate logistic regression analysis was performed using SPSS version 27, and variables with *p* < 0.05 were considered statistically significant.

**Results:**

A total of 140 neurosurgical patients were included, with an overall ICU mortality rate of 23% (*n* = 32). Emergency surgery (AOR = 7.4; 95% CI: 1.26–13.97; *p* = 0.027), intraoperative blood loss > 1000 mL (AOR = 4.0; 95% CI: 1.96–5.29; *p* = 0.044), lack of continuous postoperative monitoring (AOR = 3.5; 95% CI: 1.93–13.15; *p *= 0.044), and ICU complications (AOR = 7.8; 95% CI: 1.92–13.49; *p* = 0.004) were independently associated with increased mortality.

**Conclusion:**

23% Of the neurosurgical patients admitted to the SICU died during the ICU stay. Mortality was independently associated with emergency surgery, significant intraoperative blood loss, lack of continuous postoperative monitoring, and the occurrence of ICU-related complications.

## Background

Globally, the need for specialized medical care is changing the face of Intensive Care [7, 16]. As many neurosurgical patients require Intensive Care Unit (ICU) support during their hospital stay, treatment in specialized neurosurgical intensive care units has demonstrated clear benefits in terms of both patient management and outcomes [22, 25, 26]. An Intensive Care Unit is a specialized inpatient facility that provides continuous monitoring and care for critically ill patients, requiring advanced equipment and a multidisciplinary healthcare team. These units are extremely resource-intensive, both in terms of manpower and equipment utilization. In developed countries such as the United States, ICUs account for 15–40% of the total hospital expenditure [10, 27].

Neurosurgical (NS) intensive care includes patients with central nervous system (CNS) trauma, tumors, postoperative complications, and stroke. Systemic effects associated with neurological illnesses predispose these patients to various complications, including altered drug metabolism [4, 8]. A considerable proportion of ICU admissions consists of patients with neurosurgical conditions, including traumatic brain injury (TBI), spontaneous intracranial hemorrhage, ischemic stroke, and subarachnoid hemorrhage (SAH), and patients undergoing elective neurosurgical procedures. These individuals often require close monitoring and advanced care due to the high risk of neurological deterioration and other postoperative complications [5, 21].

Optimal postoperative care for neurosurgical patients focuses on preserving cerebral perfusion by maintaining adequate cerebral perfusion pressure (CPP) and ensuring sufficient oxygen delivery to the brain. Continuous hemodynamic monitoring and targeted interventions are required to prevent secondary brain injuries [5, 21]. Common indications for ICU admission among neurosurgical patients include the need for postoperative observation, impaired consciousness requiring close neurological monitoring, mechanical ventilation for airway protection, and occurrence of postoperative medical complications such as seizures, infections, or thromboembolic events [3, 9]. Neurosurgical conditions often require intensive postoperative care due to the complexity and critical nature of the procedures involved. Despite the increasing demand for neurosurgical services, there are limited data on the magnitude and outcomes of patients admitted to the surgical ICU. Understanding these is crucial for resource allocation, improving patient management, and reducing morbidity and mortality rates [4, 9, 21].

The absence of comprehensive data on patient characteristics, admission trends, and outcomes in the surgical ICU limits the ability of healthcare providers to optimize care for this high-risk population. Furthermore, no study has adequately described the clinical outcomes of neurosurgical patients in our setting [5]. This study aimed to explore the magnitude of neurosurgical admissions and identify the factors influencing patient outcomes in the Surgical Intensive Care Unit (SICU) of the Tikur Anbessa Hospital.

## Methodology and materials

### Study area and design

This study was conducted at the Tikur Anbessa Specialized Hospital (TASH) in Addis Ababa, Ethiopia, the country’s largest national tertiary referral and teaching hospital. Affiliated with the College of Health Sciences of Addis Ababa University, TASH serves as a major center for advanced clinical care, medical training, and research, providing specialized referral services to a national catchment population of approximately 120 million people.

The Surgical Intensive Care Unit has 12 beds and 8 mechanical ventilators, and is staffed by multidisciplinary specialists and subspecialist clinicians from Anesthesiology and Critical Care, Pulmonology and Critical Care, and Neurosurgery. The unit provides critical care services to surgical patients, including those undergoing neurosurgical procedures, with 356 critically ill admissions reported by 2023.

Data were collected from January 1, 2024, to January 1, 2025. A retrospective cohort study design was used.

### Population and eligibility

Source Population: All neurosurgical patients admitted to the SICU at the TASH.

Study Population: Neurosurgical patients who met the inclusion criteria were admitted during the study period.

Inclusion Criteria: All neurosurgical patients admitted to the SICU.

Exclusion Criteria: Patients with incomplete documentation of outcomes and those admitted to the stroke unit were not eligible.

### Sampling

All eligible neurosurgical patients who were admitted to the SICU during the study period were included. Medical records were systematically reviewed to extract relevant data.

### Study variables

Dependent Variable:

- Treatment outcome of neurosurgical patients.

Independent Variables:

- Socio-demographic: Age, sex, ASA classification.
- Clinical: Comorbidities, type of neurosurgical condition, previous neurosurgery.
- Perioperative: Duration of anesthesia or surgery, type of surgery (elective vs. emergency), blood loss, transfusion, and inotropic/vasopressor support.
- ICU factors: Complications, mechanical ventilation, tracheostomy, Glasgow Coma Scale (GCS) score at admission, and length of ICU stay.

Operational definition

- **✓ ASA Physical Status Classification**: The ASA system assesses a patient’s preoperative health, ranging from ASA I (healthy) to ASA VI (brain-dead organ donor), and helps estimate the perioperative risk [2].
- **✓ Readmission**: Reentry of patients within the study period after prior discharge; only data from the most recent admission were included in the data [13].
- **✓ Complex postoperative care**: Specialized, multidisciplinary management provided to patients who require intensive monitoring, advanced clinical interventions, and extended recovery support, typically under continuous 24-h nursing supervision. This includes the need for advanced hemodynamic monitoring, airway management, and specialized pain management strategies.
- **✓ ICU-related complications:** Any unintended, harmful, or adverse occurrence that arises during a patient’s stay in the ICU, which is directly attributable to diagnostic, therapeutic, or prophylactic interventions rather than the natural progression of the underlying disease [19].
- **✓ Intraoperative Blood Loss (IBL**): The total volume of blood lost during a surgical procedure, calculated from the time of skin incision to the time of wound closure [12].
- **✓ Continuous postoperative monitoring:** Refers to uninterrupted physiological observation in the ICU using bedside monitoring systems, including electrocardiography, pulse oximetry, noninvasive or invasive blood pressure monitoring, and regular neurological assessment (e.g., Glasgow Coma Scale). Patients were classified as having continuous monitoring when these parameters were consistently recorded during the ICU stay [11].

### Data collection tools and procedures

Structured questionnaires were adapted from a previous study. Patients’ medical records were identified from the TASH ICU logbooks. Data were collected from patients' medical records and charts. Patients’ charts were evaluated by a physician capable of recording different variables. A standardized questionnaire was used to collect data. Sociodemographic data, such as patient age, sex, ASA physical status, duration of anesthesia, and surgery, were obtained from the patient's chart and anesthesia follow-up sheet. Patterns and outcomes of neurosurgical ICU patients were measured from admission to discharge from the ICU.

### Data processing and analysis

All responses to the questionnaires were coded, entered, and analyzed using SPSS Version 27. The results of the descriptive analysis are presented in the tables and figures. Binary logistic regression analysis was employed; hence, in bivariate analysis, the variables with a *p*-value < 0.25 were candidates for multivariable analysis. To test the relationship between the dependent variables and predictors, the final results of the multivariable analysis were used, and variables with *P* < 0.05 were considered significantly associated.

## Result

### Sociodemographic characteristics of the study participants

**Fig. 1:**
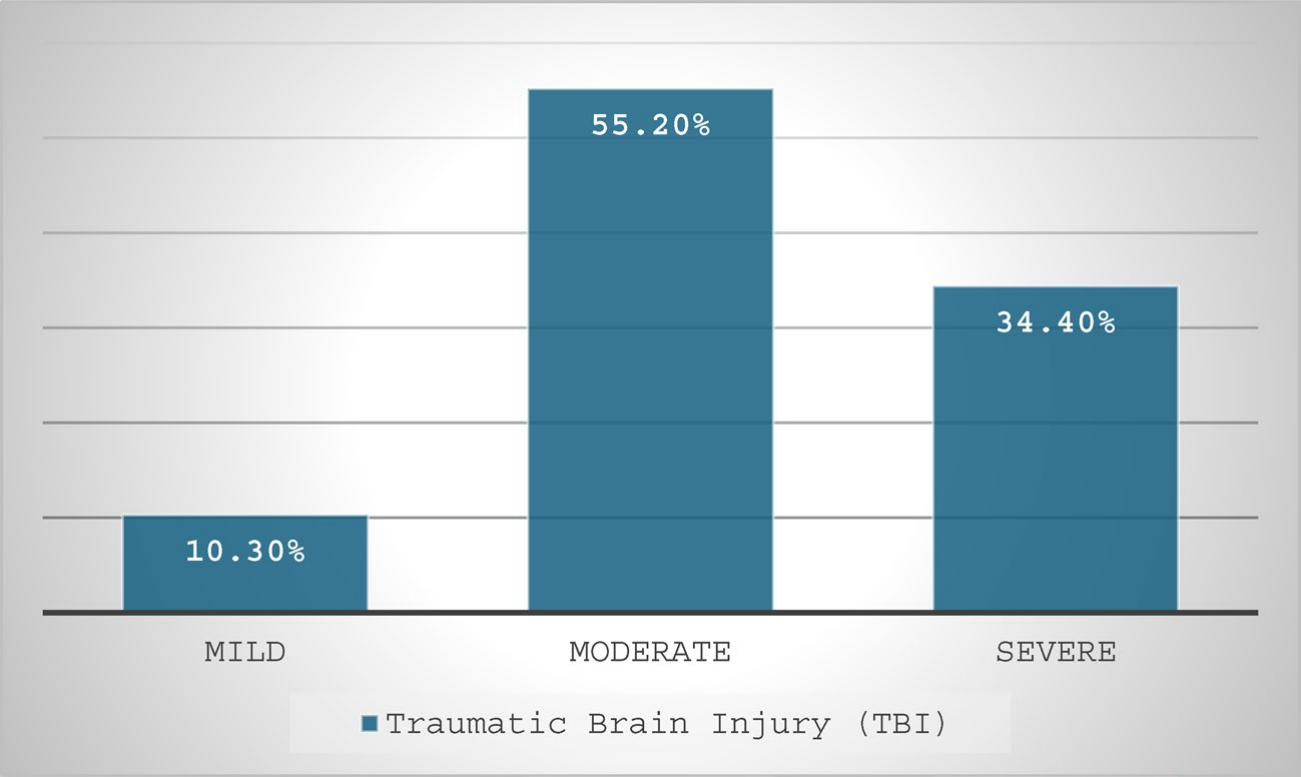
Severity of TBI patients admitted to Tikur Anbessa Hospital surgical intensive care unit from January 01, 2024, to January 01, 2025

The medical records of the eligible patients were reviewed, and the response rate was 93% (140 patients). Nearly half of the participants, 67 (47.9%), were aged 31–45 years, and 72 (51.4%) were male. One-fourth (25.7%) of the patients had at least one pre-existing medical condition, most commonly hypertension (50%), diabetes mellitus (16.7%), or seizure disorders (8.3%). Previous neurosurgical interventions were reported in 30.7% of the patients. Based on the ASA physical status classification, most participants (78.6%) were ASA II, followed by ASA I (14.3%), and ASA III–IV (7.1%). (Tables 1 and 2) (Fig. 1).

**Table 1:** Characteristics of neurosurgical patients admitted to Tikur Anbessa Hospital surgical intensive care unit from January 1, 2024, to January 1, 2025

Variable		Admission *n* = 140	
		Frequency	Percent
Age (years)	15–30	39	27.9
	31–45	67	47.9
	46–55	23	16.4
	> 55	11	7.9
Gender	Male	72	51.4
	Female	68	48.6
ASA classification	ASAI	20	14.3
	ASA II	110	78.6
	ASAIII & IV	10	7.1
Comorbidity	Yes	36	25.7
	No	104	74.3
Previous history of neurosurgery	Yes	43	30.7
	No	97	69.3
primary admission diagnosis	Brain Tumor	101	72.1
	Chronic subdural hematoma	1	0.7
	Subarachnoid Hemorrhage	7	5
	TBI	29	20.7
	Trigeminal neuralgia	2	1.4
Types of surgery	Elective	119	85
	Emergency	21	15
Types of procedure	Craniotomy and resection	83	59.3
	EVD/shunt placement	20	14.3
	Decompressive Craniotomy & Hematoma Evacuation	21	15
	Elevation of a deep skull fracture	2	1.4
	Microvascular surgery	3	2.1
	TSS	11	7.9
Intraoperative blood loss	< 500	20	14.3
	500–1000	81	57.9
	> 1000	39	27.9
Requirement of a vasopressor	Yes	18	12.9
	No	122	87.1
Blood transfusion	Yes	34	24.3
	No	106	75.7
Duration of surgery (hours)	≤ 3	35	25
	4–7	73	52.1
	> 7	32	22.9
Duration of anesthesia (hours)	≤ 3	4	2.9
	4–7	78	55.7
	> 7	58	41.4
Postoperative Neurosurgical Monitoring	Yes	101	72.1
	No	39	26.9
ICU readmission	Yes	13	9.3
	No	127	90.7
GCS and RASS score	RASS Score –2 & −3	44	31.4
	GCS < 9	10	7.1
	GCS 9 −112	24	17.1
	GCS 13–15	62	44.3
Tracheostomy done	Yes	18	12.9
	No	122	87.1
ICU complication	Yes	62	44.3
	No	78	55.7
Mechanical ventilator requirement	Yes	75	53.6
	No	65	46.4

**Table 2:** Types of comorbidities and mechanical ventilator-related characteristics of neurosurgical patients of Tikur Anbessa Hospital surgical intensive care unit from January 01, 2024, to January 01, 2025

Variable		Frequency	Percent
Types of comorbidities	Hypertension	18	50
	Hypertension and Diabetes Mellitus	4	11.1
	Diabetes Mellitus	6	16.7
	Retroviral Infection and Hypertension	1	2.8
	Epilepsy	3	8.3
	Retroviral Infection	2	5.6
	Hypertension and Epilepsy	2	5.6
	Total	36	100
Indication for MV	For a prolonged prone position	10	13.3
	Airway protection	27	36
	Respiratory Failure	10	13.4
	Delayed awakening	19	25.3
	ICP	9	12
	Total	75	100
Length of stay in MV in days	≤ 1	27	36
	2–7	27	36
	> 7	21	28
	Total	75	100

### Primary diagnosis of neurosurgical patients at admission

**Fig. 2:**
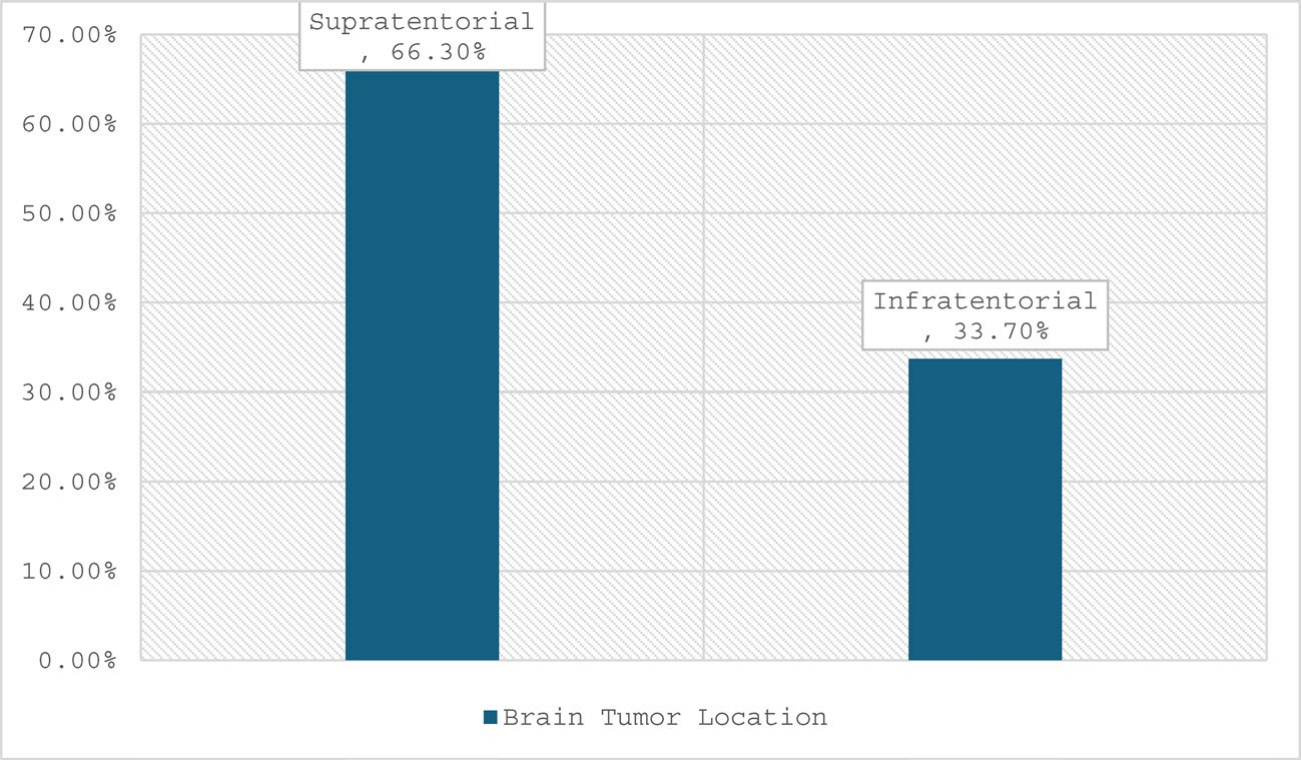
Location of brain tumor of patients admitted to Tikur Anbessa Hospital surgical intensive care unit from January 01, 2024, to January 01, 2025

During the study period, 193 elective brain tumor surgeries were performed. Of these, 101 patients (52%) were admitted to the surgical ICU and from the total study participants, 72% were admitted to the ICU with a primary diagnosis of a brain tumor (Table 1) 64.3% had supratentorial tumors, and 33.7% had infratentorial tumors (Fig. 2). TBI accounted for 20.7% of admissions, and 55.2% were in the moderate severity category (Fig. 1).

### Intraoperative-related characteristics of the study participants

Most of the surgical procedures (*n* = 119, 85%) were elective. Craniotomy and resection accounted for the largest proportion, 83 (59.3%), followed by decompressive craniotomy and hematoma evacuation, which together comprised 21 (15%) of the procedures. Intraoperative blood loss of 500–1000 mL was observed in 58% of participants, and 12.9% required vasopressor support. Blood transfusions were administered to 24% of the participants, while 52.1% underwent surgeries lasting 4–7 h. (Table 1).

### Indication of admission to the intensive care unit

**Fig. 3:**
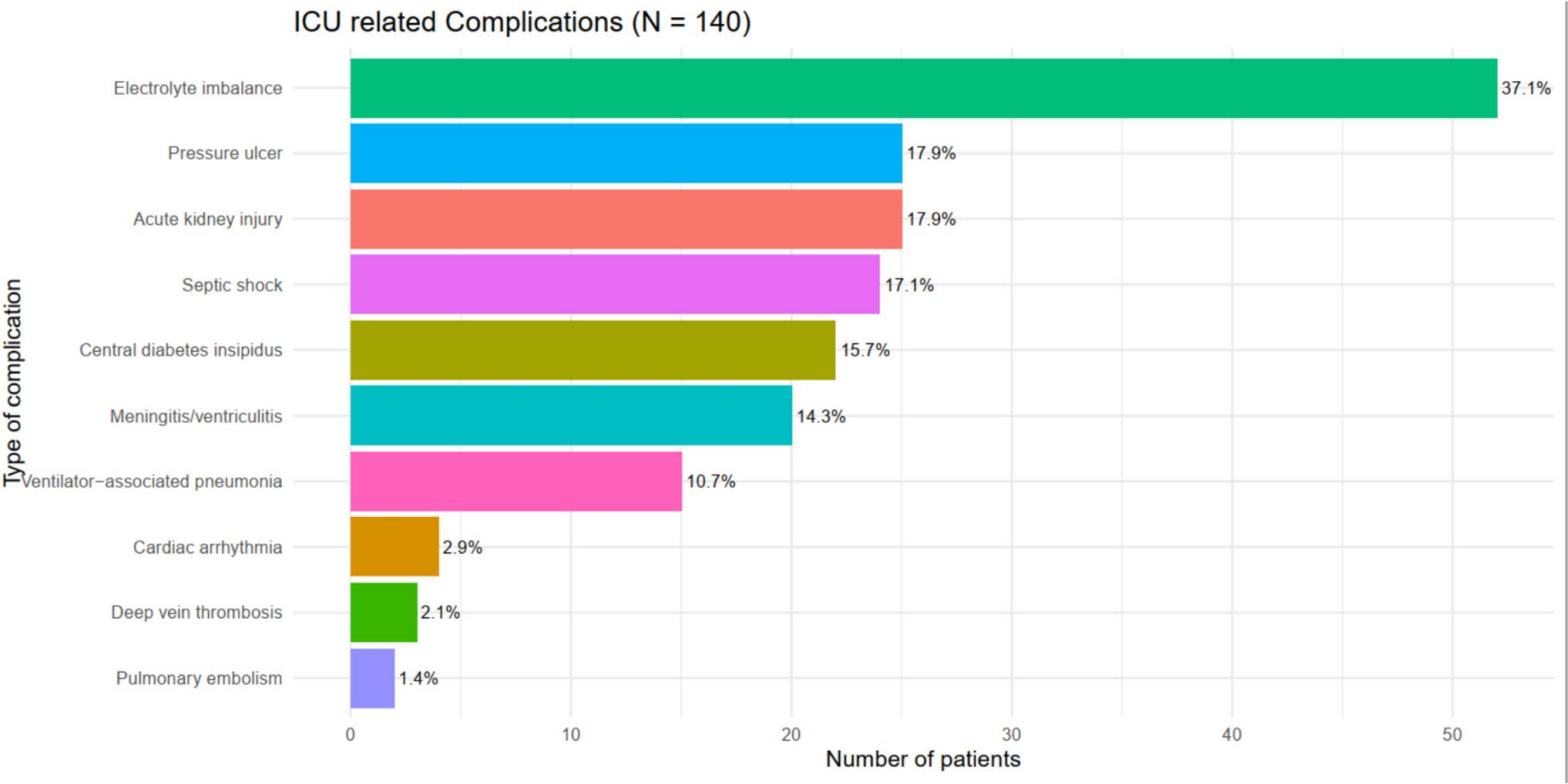
Distribution of ICU related complication in Neurosurgical patients admitted to Tikur Anbessa Hospital surgical intensive care unit from January 01, 2024, to January 01, 2025

Among the neurosurgical patients admitted to the ICU, 101 (72.1%) were admitted for postoperative neurosurgical monitoring, whereas the remaining 26.9% were admitted for other critical indications, including airway protection, respiratory failure, sepsis, and increased intracranial pressure. Of the patients admitted to the ICU, 9.3% were readmitted. Regarding neurological status, 44.3% had a Glasgow Coma Scale (GCS) score of 13–15 and 31.4% had a Richmond Agitation-Sedation Scale (RASS) score of −2 to −3. Mechanical ventilation was required in 54% of the participants (Table 1). The most frequently observed ICU complication was electrolyte imbalance (37.1%), followed by acute kidney injury (AKI) and bedsores (17.9%) and central diabetes insipidus (DI) (17.1%) (Fig. 3).

### Neurosurgical outcome-related characteristics of participants

Among patients admitted to the SICU, 22.9% died, and the remaining 77.1% were either transferred or discharged from the ICU. (Table 3).

**Table 3:** The outcome-related characteristics of neurosurgical patients admitted to Tikur Anbessa Hospital surgical intensive care unit from January 01, 2024, to January 01, 2025

Variable	Frequency	Percent
ICU Length of stay in days		
≤ 3	89	63.6
4–7	22	15.7
> 7	29	20.7
Outcome in ICU		
Deceased	32	22.9
Discharged/Transfer	108	77.1

### Determinants of mortality in the ICU of neurosurgical patients

The odds of death among patients undergoing emergency surgery were 7.4 times higher than those undergoing elective procedures (AOR 7.4, 95% CI: 1.26–13.67). Patients with intraoperative blood loss exceeding 1000 mL were four times more likely to die than those with blood loss less than 500 mL (AOR 4.0, 95% CI: 1.96–5.29). Similarly, patients admitted to the ICU for critical indications other than postoperative monitoring, such as airway protection, respiratory failure, or sepsis, had significantly worse outcomes, with an odds of death 3.5 times higher than those admitted for postoperative monitoring (AOR 3.5, 95% CI: 1.93–13.15). Additionally, patients who experienced ICU complications had 7.8 times higher odds of death than those who did not (AOR 7.8, 95% CI: 1.92–13.15) (Table 4).

**Table 4:** Determinants of mortality of patients admitted to Tikur Anbessa Hospital surgical intensive care unit from January 01, 2024, to January 01, 2025

Variable	Outcome		COR (95%CI)	*p*-value	AOR (95%CI)	*P*-value
	Death	Discharge				
Diagnosis						
Brain Tumor	23	78	0.98(0.41,2.37)	0.9		
Non-tumor admission	9	30	1			
Age in years						
15–30	9	30	1		1	
31–45	11	56	0.66(0.24, 1.76)	0.4	0.19(0.04, 0.90)	0.04
46–55	7	16	1.5(0.46, 4.65)	0.5	0.89(0.18, 4.41)	0.88
> 55	5	6	2.8(0.68, 11.28)	0.15	2.6(0.34, 19.93)	0.35
Comorbidity						
Yes	10	26	1.4(0.60, 3.45)	0.25	1.1(0.29, 4.01)	0.89
No	22	82	1		1	
Timing of surgery						
Elective	23	96	1		1	
Emergency	9	12	3.1(1.18, 8.31)	0.02	7.4(1.26, 13.97)	**0.03**
Amount of blood loss						
< 500	2	18	1		1	
500–1000	16	65	2.2(0.47, 10.54)	0.32	9.1(0.71, 17.59)	0.09
> 1000	14	25	5.0(1.02, 24.98)	0.05	4.0(1.96, 5.29)	**0.04**
Postoperative monitoring						
Yes	16	85	1		1	
No	16	23	3.7(1.61, 8.49)	0.002	3.5(1.93, 13.15)	**0.04**
Tracheostomy						
Yes	10	8	5.7(2.01, 16.04)	0.001	2.7(0.39, 18.90)	0.30
No	22	100	1		1	
ICU complication						
Yes	28	34	15.2(4.95, 46.86)	< 0.001	7.8(1.92, 13.49)	**0.004**
No	4	74	1		1	
Length of stay in ICU						
≤ 3	11	78	1		1	
4–7	5	17	2.1(0.64, 6.79)	0.22	0.74(0.17, 3.15)	0.68
> 7	16	13	8.7(3.32, 22.94)	< 0.001	1.4(0.25, 8.14)	0.69

## Discussion

In our study, neurosurgical patients accounted for 31.5% of all ICU admissions, reflecting the significant demand for critical care in this patient population. This figure is closely aligned with previous data from the Tikur Anbessa Specialized Hospital (TASH), where neurosurgical admissions were reported at 32% [6] and were comparable to the 37.9% reported in a recent study from Pakistan [23]. Remarkably, the AaBET Hospital in Addis Ababa reported the highest proportion (62.7%) due to its role as a national trauma and emergency center [15]. Other regional studies also support this trend, such as the University of Nigeria Teaching Hospital (41.2%) [18], while a tertiary ICU in Nepal reported a lower rate of 24.5%, possibly because of different case mix and referral pathways [1].

Globally, the proportion of neurosurgical ICU admissions varies widely, with national registry data from England showing a more balanced distribution across elective and emergency neurosurgical cases [28]. In Southeast Nigeria, ICU admission for neurosurgical reasons remains substantial but is influenced by the prevalence of trauma and neurosurgical workforce limitations [17]. These variations across settings highlight the importance of hospital specialization, case severity, and regional healthcare infrastructure in shaping ICU caseloads.

The ICU mortality rate among neurosurgical patients was 23% (32/140), which was consistent with the AaBET Hospital (22.9%) [15] and a study from Nepal (22%) [1]. However, this rate is higher in contrast to the lower mortality rate observed in Pakistan (6.6%) [23], Japan(14.6%)[20]and England, where elective cranial surgery mortality is as low as 0.5% and increases to 7.4% in emergency surgeries [28]. The higher mortality in our setting may reflect limitations in perioperative preparation, postoperative care, and resource availability, factors that are similar to those reported in Nigerian and Southeast Asian ICUs [17, 18].

The urgency of the surgical procedure was a key determinant of the mortality in our cohort. Patients undergoing emergency surgeries were 7.4 times more likely to die than those undergoing elective surgeries. This finding aligns with evidence from Pakistan, where 40% of neurosurgical procedures were emergency, with poorer outcomes [23], and England, where non-elective neurosurgical patients had significantly higher mortality [28].

Emergency surgeries are associated with worse outcomes, potentially reflecting higher illness severity and limited time for preoperative optimization. Greater intraoperative blood loss was significantly associated with increased mortality. In our study, patients with a blood loss of > 1000 mL had a fourfold increased risk of mortality compared to those with a blood loss of < 500 mL. Excessive bleeding contributes to hemodynamic instability and necessitates blood transfusion, which increases the risk of complications. Although several studies have not reported specific intraoperative blood loss thresholds, similar concerns regarding perioperative instability have been noted in elective neurosurgery [24].

ICU complications were common in our cohort, affecting 44.3% of patients, and were significantly associated with mortality (AOR 7.8). The most frequent complications were electrolyte imbalance (37.1%), acute kidney injury (17.9%), bedsores (17.9%), and septic shock (17.1%). These findings are similar to those of AaBET [15] and, to a lesser extent, in Pakistan, where AKI (7.1%) and arrhythmias (2%) were less prevalent owing to more robust ICU protocols [23].

The primary reason for ICU admission was postoperative neurosurgical monitoring (72.1%). However, patients admitted for nonsurgical indications such as airway protection, respiratory failure, or sepsis had significantly higher odds of mortality (AOR 3.5). This suggests that neurosurgical monitoring admissions, although resource-intensive, may have better prognoses than acute systemic illnesses requiring ICU support. Similar conclusions have been drawn from long-term observational studies, such as the Finnish neurosurgical ICU study, which showed that long-term ICU trends are shaped not only by diagnosis but also by the evolution of trauma systems and intensive care protocols [14].

Regarding airway management, our tracheostomy rate (12.9%) was between those reported in Nepal (5.5%) [1] and Pakistan (39%) [23], where early tracheostomy (median day 4) is commonly practiced. ICU length of stay and tracheostomy were significant in the univariate analysis but lost significance in the multivariable model, indicating that their association with mortality is likely confounded by the severity of illness. These factors probably reflect the underlying clinical complexity and critical disease, rather than serving as independent or causal predictors of death.

## Conclusion

Mortality was independently associated with emergency surgery, significant intraoperative blood loss, absence of continuous postoperative monitoring, and the development of ICU-related complications. These findings highlight the importance of effective perioperative optimization and vigilant postoperative monitoring in neurosurgical ICU patients. Early detection and management of ICU complications may further improve the outcomes. Strengthening surgical planning, expanding monitoring capacity, and improving critical care resources are essential strategies for reducing preventable mortality, particularly in resource-limited settings.

### Limitations of the study

This study has several limitations that should be considered when interpreting the findings. First, the retrospective single-center design limits the ability to establish causal relationships, and may reduce the generalizability of the results to other settings with different patient populations or resource availability. Second, reliance on medical record reviews introduces the possibility of documentation bias and incomplete or inaccurate data capture, particularly for clinical variables and ICU complications. Third, the relatively small sample size may have limited the statistical power to detect weak associations between predictors and mortality. Finally, the analysis was restricted to in-ICU outcomes, and the lack of long-term follow-up data precluded the assessment of post-discharge morbidity and mortality, which may underestimate the true burden of adverse outcomes among neurosurgical patients.

## Data Availability

The datasets used and/or analyzed during the current study are available from the corresponding author upon reasonable request.

## Declarations

This manuscript complies with the guidelines of all authors. All the listed authors met the authorship criteria and reviewed and approved the final version of the manuscript. This work has not been previously published and is not currently under consideration by any other journal. No specific reporting checklist was used to prepare this manuscript.

## Abbreviations

*AKI*: Acute Kidney Injury
*ASA*: American Society of Anesthesiologists
*AOR*: Adjusted Odds Ratio
*CI*: Confidence Interval
*CNS*: Central Nervous System
*COR*: Crude Odds Ratio
*CPP*: Cerebral Perfusion Pressure
*DI*: Diabetes Insipidus
*EVD*: External Ventricular Drain
*GCS*: Glasgow Coma Scale
*ICP*: Intracranial Pressure
*ICU*: Intensive Care Unit
*MV*: Mechanical Ventilation
*RASS*: Richmond Agitation–Sedation Scale
*SAH*: Subarachnoid Hemorrhage
*SICU*: Surgical Intensive Care Unit
*SPSS*: Statistical Package for the Social Sciences
*TASH*: Tikur Anbessa Specialized Hospital
*TBI*: Traumatic Brain Injury
*TSS*: Transsphenoidal Surgery
